# Supplementary material for: The role of personality traits on self-medicated cannabis in rheumatoid arthritis patients: A multivariable analysis
Source: PLoS One. 2023 Jan 12;18(1):e0280219. doi: 10.1371/journal.pone.0280219 (PMC9836304; doi:10.1371/journal.pone.0280219)
Supplement: S1 Appendix — Supplementary Data. (DOCX) [file pone.0280219.s001.docx]

**Supplementary material**

**Supplementary Data**

*The Alcohol, Smoking, and Substance Involvement Screening Test (ASSIST)*

An eight–item questionnaire to identify recreational psychoactive substance use–related health risks and SUD. For this research, substances were clustered into three groups: Tobacco, alcohol, and illicit drugs. Scores are reported as low risk (ranged from 0 – 3 for tobacco and illicit drugs, and 0 – 10 for alcohol consumption), moderate risk (ranged from 4 – 26 for tobacco and illicit drugs, and 11 – 26 for alcohol consumption), or high risk (defined as ≥27 for all substances). A higher category means a pattern of more frequent use. The presence of SUD as an exclusion criterion was defined if patients scored in the high–risk category.

*Routine Assessment of Patient Index Data 3 (RAPID–3)*

Includes three measures of RA disease activity/severity: Physical function, pain, and a patient global estimate evaluation. It has a raw score of 0 – 30, with higher scores translating into higher disease activity/severity. Four proposed categories are defined based on 0 – 30 scale cut–offs: ≤3 near–remission, 3.1 – 6.0 low disease activity/severity, 6.1 – 12.0 moderate disease activity/severity and >12 high disease activity/severity.

*Quality of life in Rheumatoid Arthritis (QoL–RA)*

An eight–item self–administered measure for QoL in an RA–specific context. Evaluated dimensions include pain, physical function, and illness perception, as well as more general dimensions including health, social interaction, family support, and emotional wellbeing. No cut–off points have been established. Higher scores indicate better QoL.

*Patient Health Questionnaire–9 (PHQ–9)*

A nine–question instrument used for screening and measuring the severity of depression. Each question is scored from 0 (not at all) to 3 (nearly every day), and higher scores translate to more severe symptoms. The total score is the sum of all items and ranges from 0 to 27. A PHQ–9 score ≥10 has a sensitivity and specificity of 85% each, for major depression.

*Generalized Anxiety Disorder–7 (GAD–7)*

A seven–item questionnaire used as a screening tool and severity measure for GAD. Each question is scored from 0 (not at all) to 3 (nearly every day). The total score ranges from 0 – 21 with higher scores translating into more severe anxiety. When used as a screening tool, further evaluation is recommended when the score is ≥10, indicating moderate anxiety disorder (sensitivity of 89% and a specificity of 82% for GAD).

*Clinical Global Impression–Severity Scale (CGI–S)*

The CGI–S provides an assessment of the clinician’s view of the patient’s illness severity. It is measured according to a seven–point scale used to rate the patient's illness at the time of assessment, which is relative to the clinician's experience with patients who have the same diagnosis. Higher number categories translate into greater illness severity, and a score of 0 translates to “not evaluated”. A current severe psychiatric disorder for exclusion criteria was defined when the CGI–S score was ≥6.

*Big Five Inventory (BFI)*

A 44–item inventory answered with a five–point Likert scale that measures an individual´s personality traits according to the Big Five theory of personality. For each dimension/trait, scores range from one to five and higher scores are indicative of higher levels for that trait. There are no published norms for BFI score comparison, but some analysis methods have been proposed.

*ICAM–Q (International Complementary and Alternative Medicine Questionnaire)*

The original version includes four main sections. For the current study, we used section 3, which is related to “consumption of medicinal products based on herbs, vitamins, minerals, or homeopathic medicines”, and was modified to assess MCU exclusively. Patients were asked about MCU (smoked, topic, or oral) in the past 12 months, as well as the main reason for its use and how helpful they found the product, if applicable. We complemented the information with a locally developed survey related to CAM use and assessed timing and motivations for MCU related to RA diagnosis and Institutional health care (7 items), source of information regarding MCU (1 item), and finally patient's perceptions of alternative medicine disclosure alongside actual MCU disclosure with the rheumatologist, and satisfaction with the medical attention (six items).

*Charlson Comorbidity Index*

It is a 19–item diagnosis–based index that has been validated in different patient populations. It assesses comorbidity dichotomously (presence/absence). It has four sections that ascend in diagnosis severity. Higher sections weight more in total score. Total score is the sum of all item scores. Higher scores translate into greater comorbidity and worse prognosis. It has shown a good ability to predict morbidity–related outcomes, such as mortality.

**Supplementary tables**

**Supplementary Table 1** – Personality scores according to data related to Medicinal Cannabis Use

|  | **Perceived beneficial effect of MCU** | | | |  | **MCU disclosure** | | | |
| --- | --- | --- | --- | --- | --- | --- | --- | --- | --- |
|  | No benefit  (n=9) | At least some benefit  (n=43) | t–score | p–value |  | No  (n=43) | Yes  (n=10) | t–score | P–value |
| Extraversion score | 2.90±0.35 | 3.04±0.31 | –1.18 | 0.24 |  | 3.02±0.33 | 3.02±0.29 | –0.01 | 0.98 |
| Agreeableness score | 3.18±0.50 | 3.20±0.38 | –0.11 | 0.91 |  | 3.17±0.39 | 3.3±0.40 | –0.90 | 0.36 |
| Conscientiousness score | 2.62±0.43 | 2.93±.50 | –1.69 | 0.09 |  | 2.85±0.52 | 3.05±0.35 | –1.13 | 0.25 |
| Neuroticism score | 3.33±0.29 | 3.53±0.33 | –1.68 | 0.09 |  | 3.49±0.34 | 3.43±0.37 | 0.45 | 0.64 |
| Openess to experience score | 2.96±0.59 | 3.21±0.39 | –1.54 | 0.12 |  | 3.14±0.46 | 3.24±0.36 | –0.61 | 0.54 |

Note: Two–tailed Student’s t–test for independent samples.

**Supplementary Table 2** – Personality scores according to low vs moderate risk in substance use

|  | **Risk in Tobacco use** | | | |  | **Risk in Alcohol use** | | | |
| --- | --- | --- | --- | --- | --- | --- | --- | --- | --- |
|  | Low  (n=164) | Moderate  (n=16) | t–score | p–value |  | Low  (n=173) | Moderate  (n=7) | t–score | P–value |
| Extraversion score | 3.01±0.29 | 3.03±0.39 | –0.26 | 0.79 |  | 3.01±0.31 | 3.03±0.18 | –0.20 | 0.83 |
| Agreeableness score | 3.16±0.36 | 3.12±0.50 | 0.40 | 0.68 |  | 3.15±0.38 | 3.33±0.23 | –1.20 | 0.22 |
| Conscientiousness score | 2.95±0.41 | 2.78±0.45 | 1.53 | 0.12 |  | 2.93±0.42 | 2.98±0.28 | –0.29 | 0.76 |
| Neuroticism score | 3.48±0.34 | 3.35±0.27 | 1.52 | 0.12 |  | 3.46±0.34 | 3.60±0.27 | –1.04 | 0.29 |
| Openess to experience score | **3.00±0.41** | **3.31±0.41** | **–2.89** | **0.004** |  | 3.02±0.42 | 3.12±0.37 | –0.61 | 0.54 |

Note: Two–tailed Student’s t–test for independent samples. Not enough observations for the “other drug use” category (not shown). P values <0.05 are boldfaced. “Other drug use” could not be computed due to no observations in its moderate risk.
